# The Long March: A Sample Preparation Technique that Enhances Contig Length and Coverage by High-Throughput Short-Read Sequencing

**DOI:** 10.1371/journal.pone.0003495

**Published:** 2008-10-22

**Authors:** Katherine Sorber, Charles Chiu, Dale Webster, Michelle Dimon, J. Graham Ruby, Armin Hekele, Joseph L. DeRisi

**Affiliations:** 1 Department of Biochemistry and Biophysics, University of California San Francisco, San Francisco, California, United States of America; 2 Biological and Medical Informatics Program, University of California San Francisco, San Francisco, California, United States of America; 3 Department of Microbiology and Immunology, University of California San Francisco, San Francisco, California, United States of America; 4 Division of Infectious Diseases, Department of Medicine, University of California San Francisco, San Francisco, California, United States of America; 5 Howard Hughes Medical Institute, University of California San Francisco, San Francisco, California, United States of America; Louisiana State University, United States of America

## Abstract

High-throughput short-read technologies have revolutionized DNA sequencing by drastically reducing the cost per base of sequencing information. Despite producing gigabases of sequence per run, these technologies still present obstacles in resequencing and *de novo* assembly applications due to biased or insufficient target sequence coverage. We present here a simple sample preparation method termed the “long march” that increases both contig lengths and target sequence coverage using high-throughput short-read technologies. By incorporating a Type IIS restriction enzyme recognition motif into the sequencing primer adapter, successive rounds of restriction enzyme cleavage and adapter ligation produce a set of nested sub-libraries from the initial amplicon library. Sequence reads from these sub-libraries are offset from each other with enough overlap to aid assembly and contig extension. We demonstrate the utility of the long march in resequencing of the *Plasmodium falciparum* transcriptome, where the number of genomic bases covered was increased by 39%, as well as in metagenomic analysis of a serum sample from a patient with hepatitis B virus (HBV)-related acute liver failure, where the number of HBV bases covered was increased by 42%. We also offer a theoretical optimization of the long march for *de novo* sequence assembly.

## Introduction

DNA sequencing technology has benefited from tremendous progress over the past several years, with many platforms routinely producing >10^9^ nucleotides (nt) of data during a single run [1]. Current generation high-throughput sequencers require a library of amplicons from which reads are generated at random by a variety of different methods, including pyrosequencing [2], reversible chain-terminator extension [3], and ligation [4]. Many of these strategies produce relatively short reads, in the range of 36–70 nt [5], compared to traditional Sanger sequencing which routinely produces reads >800 nt in length [6], [7]. For some applications, such as microRNA analysis [8], ChIP-Seq [9], or SAGE (Serial Analysis of Gene Expression) [10], short reads are sufficient. However, for resequencing known genomes [5] and *de novo* assembly of unknown sequences [11], [12], short reads present a bioinformatics challenge and make sufficient target sequence coverage difficult to achieve.

To date, experimental solutions to these difficulties have focused on two approaches: increasing the number of reads produced from a sample or extending read length. Technical advances such as paired-end reads [13], [14] or optimization of sequencing platforms with hardware, software, and/or reagent upgrades can increase the number of reads produced from a sample. Alternatively, additional reads can be produced by simply sequencing a sample multiple times. However, reaching satisfactory coverage of target sequences with these solutions is expensive.

Coverage with short-read technologies can also be increased by directly extending read length, which is achieved by increasing the number of synthesis or ligation cycles performed during sequencing. While lengthening reads does not necessarily incur additional cost, in practice, the signal to noise ratio of current technologies decreases at each cycle much more rapidly than in traditional Sanger sequencing, effectively limiting the number of bases that can be read with an acceptable degree of accuracy [3], [15].

We describe and demonstrate here a simple method for improving high-throughput short-read sequencing results using a cost-effective sample preparation technique. This process, termed the “long march,” utilizes a Type IIS restriction enzyme that cleaves DNA distal to its recognition motif [16], [17]. By embedding this recognition motif in the sequencing primer adapter of the initial amplicon library, iterative rounds of digestion and ligation produce a nested set of sub-libraries for sequencing. While we demonstrate this method using the Illumina (Solexa) GA2 platform, the long march procedure is applicable to any short-read shotgun sequencing system, including the ABI SOLiD and Helicos. We show that the long march increases contig length and absolute coverage (compared to the same number of reads produced without the procedure) using a cDNA library generated from *Plasmodium falciparum*, the protozoan parasite responsible for the most deadly form of human malaria. In addition, we show that the long march can aid in metagenomic analysis of a complex clinical specimen by increasing coverage of a particular pathogen (in this case hepatitis B virus, or HBV, in a serum sample from a patient with acute liver failure) [18]. Finally, we provide a theoretical framework for optimizing the long march for *de novo* genome assembly applications, based on relative enzyme efficiencies as well as starting DNA pool complexity. These results suggest that considerable improvements in absolute base coverage may be achieved through relatively simple and cost-effective modifications of high-throughput sequencing sample preparation protocols. In essence, the long march technique combines the desirable aspects of both shotgun sequencing and directed primer walking to produce substantially greater coverage within the same number of reads and using the same read length.

## Materials and Methods

### Long marching and barcoding bead-bound cDNA

For *Plasmodium falciparum*, 40 µL bead-bound cDNA aliquots (see Materials and Methods S1) were digested in 1× Fermentas Buffer B and 0.01 mM S-adenosylmethionine with 5 U GsuI (Fermentas International Inc., Burlington, Ontario) for 1 hour at 30°C, then at 65°C for 20 min. The digestion reactions were dephosphorylated as described in Materials and Methods S1, then washed and ligated to adapter “Sol-L-AA-NN” (short-SolL-GsuI-AANN and Sol-Adapter-L-short-phos-AA annealed). All primer sequences can be found in Table S1. Bead aliquots were again washed and resuspended in ddH_2_O. 40 µL was removed for PCR amplification with fullModSolS and Sol primer 1 for 10 cycles (see Materials and Methods S1 for PCR conditions). The remaining 2 aliquots were digested again with GsuI, dephosphorylated, washed, and ligated to adapter “Sol-L-CC-NN” (short-SolL-GsuI-CCNN and Sol-Adapter-L-short-phos-CC annealed). After ligation, the beads were again washed and resuspended, and 40 µL was removed for PCR amplification with fullModSolS and Sol primer 1 for 10 cycles, while the remaining beads underwent one more round of GsuI digestion, dephosphorylation, washing, and ligation to adapter “Sol-L-TT-NN” (short-SolL-GsuI-TTNN and Sol-Adapter-L-short-phos-TT annealed). The final aliquot was washed after ligation and PCR amplified with fullModSolS and Sol primer 1 for 10 cycles.

For the HBV sample, the long march and barcoding were carried out in an essentially identical fashion to that of *Plasmodium falciparum* with the following modifications: (1) the HBV sample used the adapters “Sol-L-CC-RR” (short-SolL-GsuI-CCRR and Sol-Adapter-L-short-phos-CC annealed), “Sol-L-GG-RR” (short-SolL-GsuI-GGRR and Sol-Adapter-L-short-phos-GG annealed), and “Sol-L-TT-RR” (short-SolL-GsuI-TTRR and Sol-Adapter-L-short-phos-TT annealed) for march rounds 1 through 3, and (2) PCR amplification of all marched aliquots was carried out for 15 cycles instead of 10 cycles using the PCR conditions described for the initial HBV library in Materials and Methods S1.

### Solexa sequencing of initial and long marched cDNA

For *Plasmodium falciparum*, the initial library and each marched sub-library were clustered on a Solexa flow cell in a separate lane (Illumina, Hayward, CA). For the HBV sample, the initial library and round 3 marched sub-library were clustered with 15 other barcoded clinical samples in one lane. Following cluster generation, Sol-SeqPrimer was annealed to the clusters on the flow cell, and 48 cycles (*P. falciparum*) or 36 cycles (HBV) of single base pair extensions were performed with image capture using an Illumina (Solexa) GA2 sequencer (Illumina, Hayward, CA). The Solexa Pipeline software suite version 0.2.2.6 (Illumina, Hayward, CA) was utilized for base calling from these images. Base called data can be found at http://derisilab.ucsf.edu/data/longmarch.

### Analysis of sequence data

Illumina's Solexa software ELAND was used to align reads, with the initial two nt of marched sub-library reads masked, to either *Plasmodium falciparum* genome release 5.4 [23] or to the HBV genome (accession number: NC_003977) [21]. Any reads that did not match the genomes in a unique position were not considered for further analysis. Genome-aligned reads that mapped to the same genomic coordinates were then collapsed into one to determine the redundancy of each library.

The percent of *P. falciparum* reads converted to the destination barcode for each round was determined by examining the initial two barcoded nt of the full reads in each lane. For reads with the correct barcode, if the barcode did not match the two bases directly upstream of the genomic alignment, it was considered “definitely barcoded.” If the barcode did match the two bases directly upstream of the genomic alignment, it was considered “possibly barcoded.” The ratio of “definitely barcoded” reads to total reads was calculated as a conservative estimate of barcoding efficiency for each library. The number of “definitely barcoded” reads, plus the number of “possibly barcoded” reads times the barcoding efficiency, gave the estimated number of correctly barcoded reads due to ligation. This number divided by the total number of reads gave the estimated percent of correctly barcoded reads resulting from ligation.

The offset histogram was calculated by comparing the starting positions of the *P. falciparum* reads in each dataset. For the march round 3 line, the upstream reads were half of the location-collapsed reads with no barcode (NN) from the initial library lane and the downstream dataset was an equal number of location-collapsed reads with a TT barcode from the lane marched three times. For the initial library line, half the location-collapsed reads with no barcode (NN) from the initial library lane were compared with the other half. The offset was counted as the distance from the start of the upstream read to the start of the downstream read.

Contig length for *P. falciparum* was calculated by counting the length of genomic segments covered by at least one read for 400,000 randomly selected reads from the initial library and the round 3 sub-library. Contig lengths were then averaged independently for each library.

### Calculation of genome coverage

For both *P.falciparum* and HBV sample libraries, reads from the initial and the round 3 libraries were chosen at random to fill datasets of various fixed sizes. Each dataset was then mapped back to its respective genome (minus the first 2 nt) and the number of genomic bases covered was determined. In order to account for extremely small dataset sizes, HBV datasets were randomly filled and analyzed 1000 times and the coverage results were averaged.

### Simulating optimization of the long march for *de novo* genome assembly

The theoretical probability of a contig-generating match between two sequences (p_m_) was calculated as a function of the overlap length between the sequences (O_L_). Equal probability of all four nucleotides at each position was assumed. The p_m_ value was taken as the number of matching sequences (s_m_) divided by the number of total sequences (s_t_) of length O_L_. When only perfect matches were considered, s_m_ = 1 and s_t_ = 4^∧^O_L_, so p_m_ = 1/4^∧^O_L_. When mismatches were allowed, s_m_ equaled the number of sequences within the allowed mismatch distance, which was calculated as described [24]. Given a dataset of S unique sequences, the probability of a sequence being spuriously joined with another to form a contig (p_s_) was calculated as p_s_ = 1−(1−p_m_)^∧^S. The probability of at least one sequence in a dataset of size S being spuriously linked to another (p_st_) was calculated as p_st_ = 1−(1−p_s_)^∧^S. The assumption of a search for overlap between the 3′ end of the given read and the 5′ ends of the remaining reads was assumed when calculating p_s_. Therefore, the value of p_st_ reflected the application of p_s_ to an all-against-all search in which each sequence could be connected to all others based on either a 5′ overlap, a 3′ overlap, or both.

Assembly was simulated *in silico* using an abstract amplicon data class. Each amplicon contained a number of step positions numbered from zero through the number of simulated march rounds. A number of amplicon instances was created equal to the simulated amplicon pool complexity. The number of reads obtained was specified for each simulation. For each read, an amplicon instance was selected randomly (assuming evenl representation of all amplicons in the pool), and a step number was randomly selected for that amplicon with the probabilities of various steps weighted as specified. The resulting amplicon-step combination (read) was added to a collection, and the contents of that collection were evaluated in terms of the redundancy of its contents and the ability to assemble amplicon sequences. Reads were joined into a contig if they derived from adjacent step positions of the same amplicon instance. Unlinked reads formed contigs of length = 1.

## Results

### The long march uses a Type IIS restriction enzyme to create a series of nested sub-libraries with reduced read redundancy

The long march approach exploits the ability of certain classes of restriction enzymes (Type IIS and some Type III enzymes) to cleave DNA downstream of their recognition motifs [19]. These motifs are engineered into the required library adapters to permit iterative rounds of restriction enzyme cleavage and adapter ligation, which produce a set of nested sub-libraries. One can sequence either the sub-library generated at the final round or a combined pool created by mixing successive sub-libraries, depending on the efficiency of cleavage and ligation during the long march.

**Figure 1:**
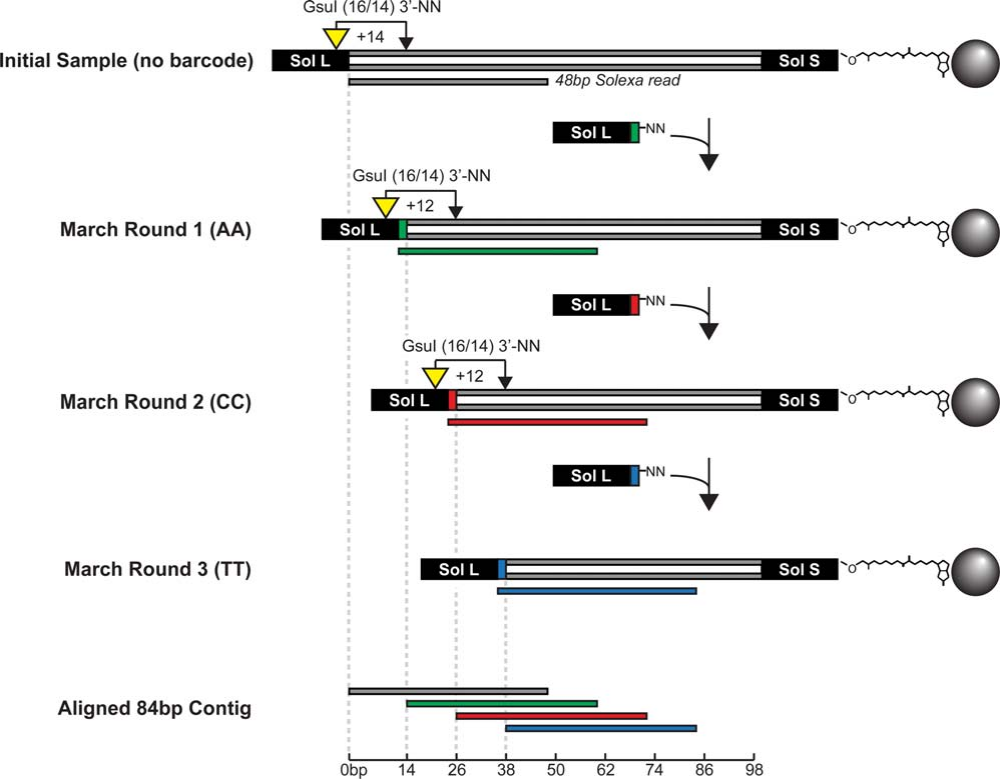
Iterative rounds of GsuI digestion and barcoded adapter ligation create nested sub-libraries. Adapter flanked cDNA molecules are attached to streptavidin beads via biotin modification of the Sol-S adapter. Yellow triangles indicate the GsuI recognition motif engineered into the Sol-L adapter, while the connected black arrow represents the distal cut site. Adapter barcodes and corresponding reads are classified as AA (green), CC (red), or TT (blue). Reads from the initial library and all three long march steps are aligned to form an 84 bp contig.

To initiate the long march procedure, RNA from *Plasmodium falciparum* was reverse transcribed into double-stranded cDNA, biotinylated, and bound to streptavidin beads (see Materials and Methods S1). In construction of the initial library, the adapter containing the sequencing primer hybridization site (Sol-L) was modified before its NN overhang to incorporate the recognition motif of the Type IIS restriction enzyme GsuI (5′-CTGGAG-3′). Each march round began with digestion of the bead-bound cDNA with GsuI, which cleaves double-stranded DNA 14 nt distal to this motif (Figure 1) [16], [17]. Digested cDNA was then ligated to barcoded Sol-L adapters, and this digestion and ligation process was repeated iteratively to generate three nested sub-libraries in addition to the initial cDNA library. The initial library contained no barcode while subsequent rounds were barcoded AA, CC, and TT, respectively. After 5–10 cycles of PCR, the initial library and each sub-library was clustered and sequenced in a separate Illumina (Solexa) GA2 flow cell lane.

The resulting 48 bp sequence reads were aligned to the *P. falciparum* genome (23 Mb) using Illumina's ELAND software [20]. This analysis yielded the working dataset of genome-aligned reads presented in Table 1 and all subsequent analysis is based on this dataset unless otherwise noted.

**Table 1:** Overview of sequencing reads obtained for each sample.

Sample	Library	Total Reads*	Genome-Aligned Reads (% of Total Reads)	Location-Collapsed Reads (% of Genome-Aligned Reads)
*P. falciparum*	Initial Library	2,316,937	525,509 (22.7%)	134,912 (25.7%)
	Round 1	4,194,002	968,063 (23.1%)	308,173 (31.8%)
	Round 2	2,747,609	485,034 (17.1%)	200,754 (41.4%)
	Round 3	4,881,843	1,088,583 (22.3%)	415,836 (38.2%)
HBV	Initial Library	294,625	328 (0.1%)	94 (28.7%)
	Round 3	643,611	1291 (0.2%)	416 (32.2%)

* *Plasmodium falciparum* reads are 48 bp long, while HBV reads are 36 bp long.

In order to estimate the redundancy of each library, reads aligned to the genome were collapsed by location–that is, reads that mapped to the same genomic coordinates were merged into one. Location collapse was used rather than sequence-based collapse to discount aligned reads with sequencing errors. While the genome-aligned reads from the initial library collapsed to 25.7% of the original dataset (an average of 3.89 reads collapsed into one), the genome-aligned reads from the round 3 sub-library collapsed less, to 38.2% of the original dataset (an average of 2.62 reads collapsed into one) (Table 1). These results indicate that the long march reduced the redundancy of the initial cDNA library.

### Marching creates offset overlapping reads and longer average contigs

**Figure 2:**
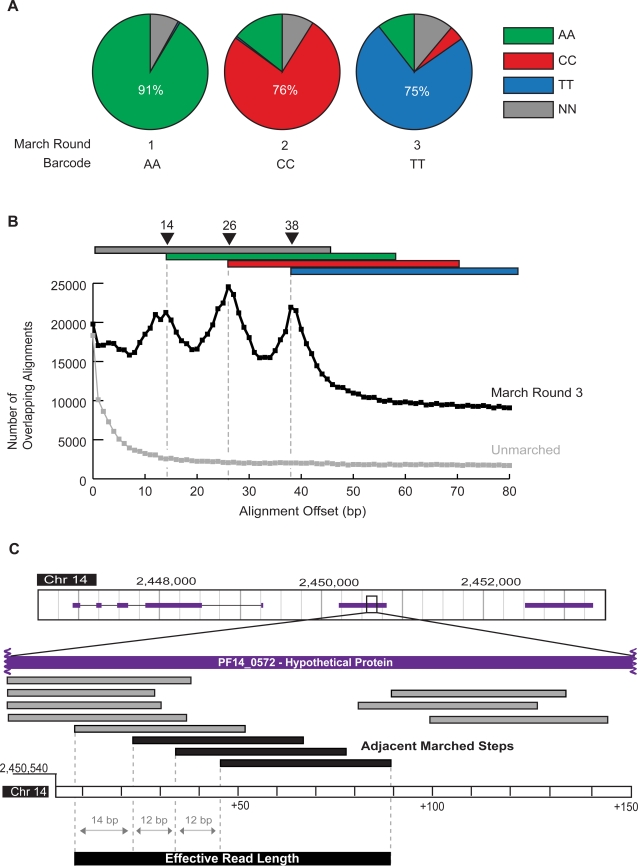
The long march produces barcoded, offset reads that aid in contig growth. (A) Barcodes for each round of the long march. The first two bases, masked during genomic alignment, were analyzed for all reads aligning to the *P. falciparum* genome. Barcodes are classified as AA (green), CC (red), TT (blue) and NN (gray), where NN represents any barcode other than AA, CC, or TT. For each round of marching, the dominant barcode was that of the adapter added during that round. (B) Histogram of offset, overlapping alignments between 400,000 reads from the round 3 sub-library and 400,000 reads from the initial library. Reads were aligned to the *P. falciparum* genome and the difference between the starting positions of their 5′ termini was measured in cases where a round 3 read mapped distal to an initial library read. The resulting three peaks represent reads successfully marched once, twice, or three times. The gray line demonstrates that similar analysis of two pools of 400,000 reads from the initial library show no offset peaks. (C) Example of contig joining by adjacent marched reads from the same amplicon. A segment of *P. falciparum* chromosome 14 from 2,450,540 to 2,450,690 (representing a portion of the “hypothetical protein” gene PF14_0572) demonstrates the long march's utility in increasing contig size. Reads from all four libraries mapping to the area are shown. The four bottom reads derive from the libraries marched zero, one, two, and three times, respectively. While the gray reads cover much of the region shown, the adjacent marched steps from the last gray amplicon, shown in black, are required to cover the entire area and stitch together neighboring contigs.

The first two nucleotides of each read from the three *P. falciparum* sub-libraries were analyzed to determine the fraction of reads in each pool that successfully ligated to the appropriate barcoded adapter (Figure 2A). The first round of digestion and ligation, which should have added an AA barcode to each cDNA molecule, resulted in 91% of sequenced reads possessing an AA barcode. After adjusting for reads beginning with AA by chance instead of by ligation, we estimated that 89% of reads from the first round of marching received a barcoded adapter (see Materials and Methods). The second round of marching resulted in 76% CC barcodes (∼76% from barcoded adapter ligation), while the third round of marching resulted in 75% TT barcodes (∼71% from barcoded adapter ligation). The high percentage of correctly barcoded reads from each marched sub-library confirms that significant decreases in digestion and ligation efficiency did not occur over three rounds of the long march procedure.

Successful ligation of the barcoded adapters to each sub-library does not necessarily indicate that amplicons were iteratively marched forward. To assess how well the long march succeeded in producing offset, overlapping reads along library amplicons, the genome locations of successfully barcoded reads from the final round of digestion and ligation and non-barcoded reads from the initial library were compared. In cases where a read from the final round mapped downstream of a read from the initial library, the distance between the 5′ termini was measured (Figure 2B). In an ideal long march, where both digestion and ligation efficiency are 100%, this comparison would yield a histogram of alignments with one offset peak at 38 bp (14 bp+12 bp+12 bp) corresponding to molecules three steps removed from the original amplicon. While GsuI cuts 14 bp into the cDNA [16], [17], the portion removed in rounds 2 and 3 contained a two nucleotide barcode that did not match the genome, thus reducing the effective offset to 12 bp for those rounds. However, because the efficiency of each round was not 100%, three peaks emerged, representing cDNA that was successfully digested and ligated once, twice, or all three times (Figure 2B). The first (14 nt) and second (26 nt) offset peaks each displayed a distinct shoulder two nucleotides 5′ of the expected peak, because some molecules were not successfully ligated to the unbarcoded adapter initially but were later ligated to barcoded adapters, leading to a first step of 12 bp, rather than 14 bp. To control for chance offset unrelated to the long march protocol, the same analysis was performed comparing half of the reads from the initial library to the other half. This analysis yielded no offset peaks, indicating that the long march procedure was responsible for the peaks observed at 14 bp, 26 bp, and 38 bp.

The ability to construct long contigs is important in both resequencing and *de novo* assembly applications. Therefore, the average contig sizes for the initial and the round 3 libraries were calculated using 400,000 reads each. Contigs were defined as continuous stretches of the *P. falciparum* genome covered by at least one read. The long march procedure increased the average contig size from 59 nt to 69 nt. In addition, the long march resulted in more exceptionally long contigs due to its ability to connect shorter contigs by covering previously inaccessible intervening sequence. The final sub-library generated 17 contigs >1000 nt, the longest of which was 4952 nt, whereas the initial library generated only 7 contigs >1000 nt, the longest of which was 1630 nt. Library coverage for PF14_0572 (a “hypothetical protein” gene located on the minus strand of chromosome 14 from nt positions 2,450,143 to 2,450,743) demonstrated the benefit to contig assembly provided by the long march (Figure 2C). Without the series of overlapping marched reads indicated at the bottom, the region from 2,450,594 to 2,450,621 remained unsequenced and the contigs on either side were discontinuous. However, the additional information gained from sequencing these adjacent marched reads covered the previous gap and stitched the two contigs together into a much longer total covered area.

### The long march increases sequence coverage

**Figure 3:**
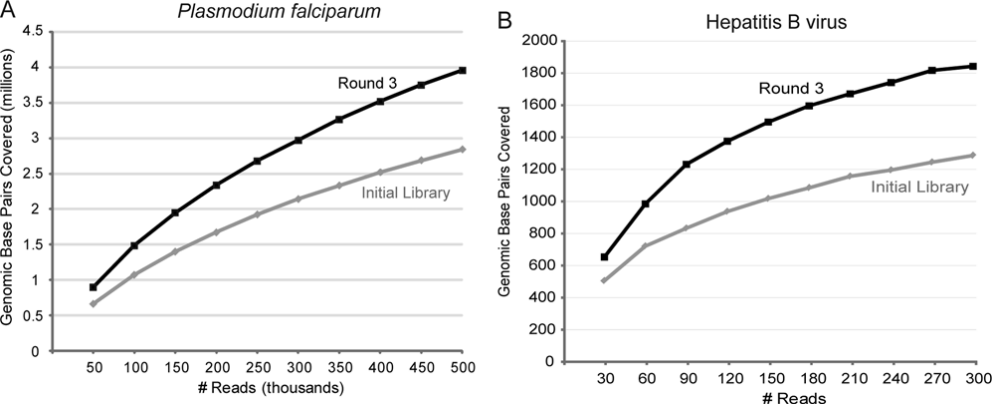
Marched sub-libraries show significantly increased genome coverage over a wide range of dataset sizes. Identical numbers of genome-aligned reads were randomly sampled from the round 3 sub-libraries and the initial libraries to simulate varying degrees of sequencing depth. The number of genomic base pairs covered by at least one read (y axis) was computed and plotted against the number of randomly selected input reads (x axis) for A) *Plasmodium falciparum* and B) hepatitis B virus (HBV) samples. Because of the small dataset sizes for HBV, each dataset of a given size was randomly filled and analyzed 1000 times; graphed coverage is an average for those datasets.

In addition to contig size, the advantage to total genome coverage provided by the long march was examined. Several datasets of randomly sampled genome-aligned reads from the round 3 sub-library and from the initial library were mapped back to the *P. falciparum* genome and the number of genomic bases covered by at least one read was measured for each dataset (Figure 3A). Even with a small dataset of 50,000 reads, the round 3 sub-library covered 35% more genomic bases (898,625 nt) than the initial library (664,114 nt). As the number of reads in each dataset grew, so too did the difference in coverage. At 500,000 reads apiece, the marched sub-library vastly outpaced the initial library by covering an additional 1.1 million bases, an increase in coverage of 39%.

The long march protocol was also applied to RNA extracted from a serum specimen from a patient with HBV-related acute liver failure (“HBV sample”) in order to assess its applicability to metagenomic analysis. 36 bp reads from the initial library as well as the round 3 sub-library were aligned to the HBV genome (3.2 kb) using ELAND (see Materials and Methods) [21]. Sequencing of the round 3 sub-library generated a greater percentage of location-collapsed HBV reads than were generated by sequencing the corresponding initial library (Table 1). This trend translated to enhanced genome coverage of HBV–with a dataset of 300 genome-aligned reads, the round 3 sub-library covered 42% more genomic bases (1828 nt) than the initial library (1284 nt) (Figure 3B). Thus the long march increases coverage of a target genome in both resequencing and metagenomic contexts.

### Simulating optimization of the long march for *de novo* genome assembly

**Figure 4:**
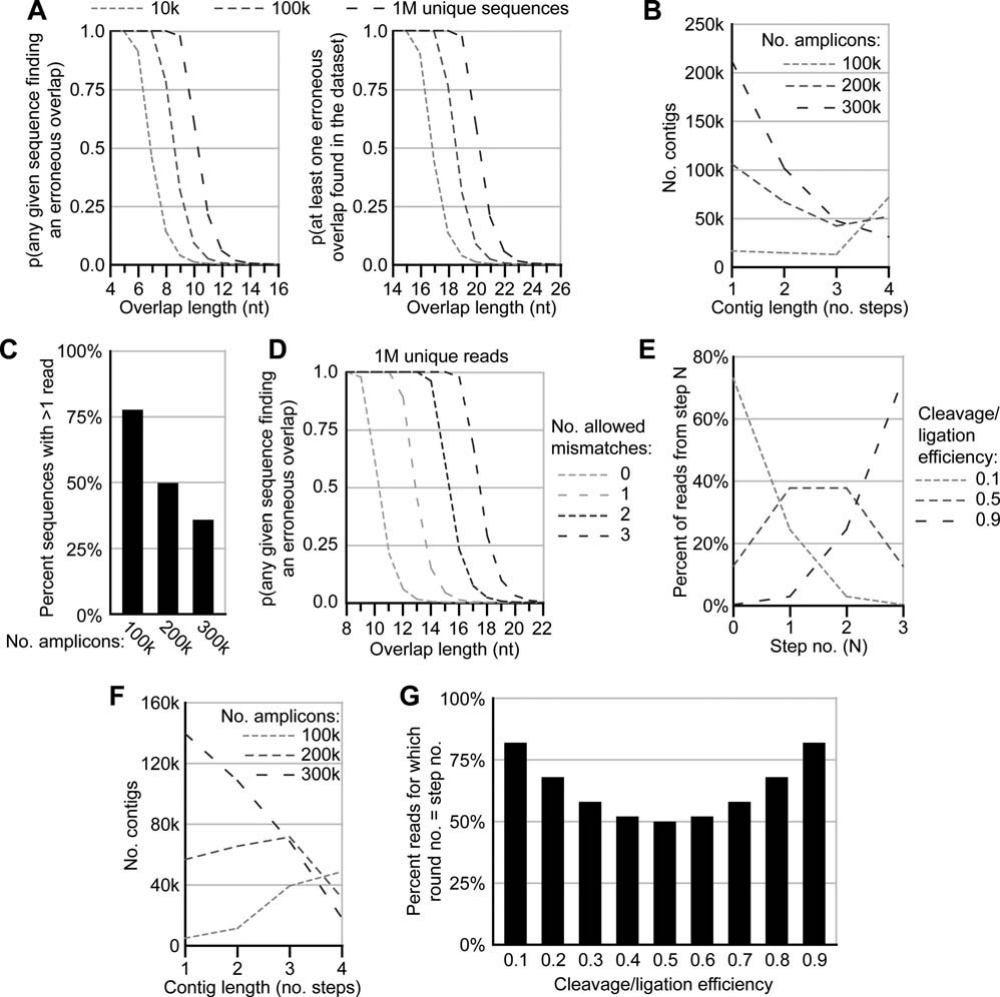
Theoretical optimization of the long march for *de novo* amplicon assembly. (A) Effect of overlap length on the probability of erroneous assembly of non-overlapping reads. For datasets with the indicated numbers of unique sequences, the probability was calculated of each sequence being erroneously joined to another in the dataset (left) or of at least one read in the dataset being erroneously joined to another (right). (B) Effect of initial pool complexity on the length of contigs. For each indicated number of amplicons in the initial pool, a simulation was performed assuming 1 million reads, and contigs were built by joining adjacent reads (see Methods). Each distribution of contig lengths, expressed in number of unique sequences assembled into the contig, was derived from a single simulation. (C) Effect of initial pool complexity on dataset redundancy. Simulations were performed as in (B) for each of the indicated amplicon pool complexities, and the fraction of unique sequences that were observed more than once is indicated. (D) Effect of allowed mismatches on the probability of erroneous assembly of non-overlapping reads. Probabilities were calculated assuming datasets of 1 million unique sequences. Allowed mismatches were single-nucleotide substitutions in the context of an ungapped alignment. (E) Effect of cleavage/ligation efficiency on the distribution of reads across the four steps of a three-round march. “Step 0” refers to unreacted molecules after three rounds of marching, while “Step 1”, “Step 2”, and “Step 3” refer to molecules that have been cleaved/ligated in one, two, or all three of three march rounds, respectively. (F) Effect of initial pool complexity on the length of contigs given a non-uniform distribution of reads across four steps. Contig lengths were determined through simulation as in (B), but using the probability of obtaining a read from each step as determined in panel (E) assuming a cleavage/ligation efficiency of 0.5. (G) Expected correspondence between round-associated barcode tags and the step no. of tagged reads. For instance, round no. = step no. = 1 if a molecule was cleaved/ligated in the first round and only the first round and was therefore tagged with the first round barcode and was advanced by one step along the amplicon template.

We used theoretical considerations to assess the utility of the long march protocol for *de novo* genome or metagenome assembly as well. For such assembly to be reliable, the length of overlap between any two reads must be sufficient to identify their common origin [22]. In the initial *P. falciparum* library, the extent of overlap between reads decayed exponentially (Figure 2B) and therefore included many instances of both insufficient overlap for *de novo* assembly and excess overlap for minimal contig extension. In the long march procedure, a step size can be selected that creates the minimum overlap between adjacent steps necessary for correct assembly given the read length and dataset size. To avoid spurious joining, datasets with many unique sequences required longer overlaps than those with few unique sequences (Figure 4A).

Modeling and simulation of the assembly process revealed amplicon library complexity to be critical to the assembly of marched reads into contigs. The benefit gained from optimization of overlap length requires the sequencing of all steps from a given library amplicon within a reasonable number of reads. With increasing complexity of the template pool, this stipulation becomes less likely. Given a dataset of one million randomly-selected reads and assuming that only adjacent steps have enough overlap to be unambiguously assembled, the majority of reads could not be joined into contigs of ≥2 steps until the pool complexity was reduced to <200,000 amplicons (Figure 4B). Reduction of pool complexity also generated higher read redundancy (Figure 4C), the error-correcting potential of which would permit lower mismatch tolerances during assembly, in turn reducing the probability of spurious joining (Figure 4D). Thus, a balance must be struck with the long march in *de novo* assembly applications between genome coverage and contig assembly.

In the above simulations, equal probability of generating a read from any step along an amplicon was assumed. However, the true distribution of sequencing substrates among march steps reflects the cleavage/ligation efficiency during the long march. In simulated sequencing of a round 3 sub-library, the calculated abundance of reads derived from the Nth step (where N can be 0, 1, 2, or 3) was biased towards high N values when cleavage/ligation efficiencies were high and towards low N values when cleavage/ligation efficiencies were low (Figure 4E). Either of these scenarios negated the benefits of marching because few adjacent steps from the same amplicon were sequenced. The most even distribution of reads along march steps was producecd with intermediate cleavage/ligation efficiencies (Figure 4E). Simulation of contig assembly using a cleavage/ligation efficiency of 0.5 resulted in fewer full-length contigs, but also fewer unjoined reads, than was produced given an artificially even distribution of reads across all march steps (Figure 4F; compare to Figure 4B).

The possibility of guiding contig assembly by applying a unique barcode to each round of marching was also considered. Such tagging would reduce the probability of misassembling reads by reducing the number of candidate reads for each step (Figure 4A), but would only be effective if reads with barcodes corresponding to the Nth march round also represented the Nth step. The failure of a molecule to cleave/ligate at one round of marching would result in the Nth step receiving a tag from round N+1 and prevent its proper assembly with reads from the N−1 step. Generally, the use of barcodes to guide assembly was not predicted to be useful due to the low frequency with which this requirement would be met, especially at the intermediate cleavage/ligation efficiencies yielding the most uniform distribution of reads across steps (Figure 4G).

## Discussion

Although the cost per base provided by short-read sequencing technologies, such as Illumina, SOLiD, and Helicos is at present far lower than longer read sequencing technologies, like 454 or Sanger sequencing, shorter read lengths pose significant challenges for resequencing and *de novo* assembly applications. The long march overcomes these challenges by extending the average contig length and significantly increasing the target sequence coverage obtained from high-throughput short-read sequencing technologies without the cost of obtaining more reads per sample or the high error rate of directly extending read lengths. High-throughput sequencing platforms generally require the addition of adapters to the ends of DNA fragments. The long march utilizes repeated cycles of Type IIS restriction enzyme cleavage and adapter ligation to allow extended sequencing of each library amplicon without loss of gene expression information. We have demonstrated the utility of the long march in the context of transcriptome resequencing (*Plasmodium falciparum*), as well as in the context of clinical specimen metagenomics (HBV). We have also provided a theoretical framework for the application of the long march to *de novo* genome assembly.

The long march protocol capitalizes on amplicon library redundancies resulting from biases introduced during sample preparation (in our case, random-primed cDNA synthesis followed by PCR library amplification) [25]. These redundancies typically result in wasteful sequencing of multiple identical short reads derived from the ends of identical amplicons. For the *Plasmodium falciparum* and HBV samples described here, the long march extended the amount of genome coverage within a dataset of a fixed number of reads, even when that dataset was relatively small. This extension in genome coverage stems from narrowing the dynamic range of individual nucleotide coverage, since redundant reads from the initial libraries were distributed over a longer distance after the libraries were marched.

In metagenomic analysis, short-read redundancy can obscure the identities of the organisms present in the sample. Characterization of microbial diversity and function from metagenomic sequence data is dependent on the identification of homology to known biological sequence [26]. Longer contigs permit more effective detection of genetic homology to known sequences by use of BLASTN or TBLASTX [27], [28]. The availability of greater coverage and longer contigs from the long march improves the likelihood of successful alignment and thus discovery of both known and novel organisms in a heterogeneous metagenomic sample.

The ability to assemble overlapping reads into reliable contigs is also crucial for *de novo* genome sequencing applications. With standard amplicon libraries, chance is relied upon to produce reads with sufficient overlap for assembly, and thus short-read datasets pose particular challenges by limiting the amount of overlap obtainable between any two reads. The long march allows read overlaps to be biased toward lengths sufficient for accurate assembly but also conservative enough to promote contig growth. Informed choice of restriction enzyme allows adjustment of the procedure's step size to facilitate accurate assembly of a predicted number of unique sequences. Also, in order to capture the adjacent march steps from a given amplicon necessary for contig assembly, library complexity, as well as cutting and ligation efficiency, must be taken into account. Reduction of library complexity may be required in order to capture enough adjacent march steps to enhance assembly within a reasonable number of reads. If a high cleavage and ligation efficiency (>80%) is achieved, bias toward sequencing only the last march steps of each amplicon can be counteracted by sequencing a pool of the marched sub-libraries from each round, rather than sequencing only the final round sub-library. However, low cleavage and ligation efficiency (<20%) cannot be overcome so easily. While low efficiencies do result in some gain in target sequence coverage (data not shown), both the restriction and ligation enzymes used for long march should be tested for robust activity before beginning the procedure.

The long march protocol described here was not optimized for a particular application. Because the long march relies only on minor modifications to adapter sequence and an appropriate Type IIS or Type III restriction enzyme, it can be readily customized for a variety of applications. Here, marching was carried out for 3 rounds; the only theoretical limit to the number of iterative rounds is the length of the starting amplicons. Also, the restriction enzyme GsuI (5′-CTGGAG-3′; 16/14) [16], [17] was chosen arbitrarily; another restriction endonuclease could be used, such as the Type III restriction enzyme EcoP151, which cleaves at a site much further downstream than GsuI (5′-CAGCAG-3′; 27/25) [29]. For these studies, long march rounds were tagged using a 2 nt DNA barcode encoded within the adapter sequence. However, the use of DNA barcodes also has the potential to allow multiple samples to be individually coded, and then sequenced simultaneously without physical separation. This approach is appropriate in applications where only a fixed depth of sequencing is required (e.g. detection of small nucleotide polymorphisms (SNPs); resequencing of small genomes or genomic subregions; pathogen detection), and/or where multiplexing of samples makes high-throughput sequencing more cost-effective.

## Supporting Information

Materials and Methods S1 Materials and methods for sample acquisition and construction of initial Plasmodium falciparum and HBV cDNA libraries.
(0.03 MB DOC)

Table S1 Primer sequences for initial library preparation and the long march.
(0.02 MB DOC)
