# Supplementary material for: The Long March: A Sample Preparation Technique that Enhances Contig Length and Coverage by High-Throughput Short-Read Sequencing
Source: PLoS One. 2008 Oct 22;3(10):e3495. doi: 10.1371/journal.pone.0003495 (PMC2566813; doi:10.1371/journal.pone.0003495)
Supplement: Materials and Methods S1 — Materials and methods for sample acquisition and construction of initial Plasmodium falciparum and HBV cDNA libraries. (0.03 MB DOC) [file pone.0003495.s001.doc]

# Materials and Methods S1

***Sample acquisition and nucleic acid preparation***

30 mL of starter culture containing 11% *Plasmodium falciparum* 3D7 Oxford highly synchronized late schizont parasites was allowed to invade 140 mL of unparasitized blood in 1 L of culture medium in a 5 L dished bottom bioreactor (Applikon Inc., Brauwegg, Netherlands). Bioreactor conditions and culture medium were as in *Bozdech et al, 2003* (1). 4 hours later, the culture was diluted with 3 L of culture medium. 7% of the starting culture was harvested 44 hours after invasion, pelleted and frozen at -80°C (1). Total RNA was harvested using Trizol (Invitrogen Corp., Carlsbad, CA), then poly-A selected using the Micro FastTrack 2.0 kit (Invitrogen Corp., Carlsbad, CA).

The clinical sample used in this study (“HBV sample”) was a serum sample from a patient presenting with acute liver failure (ALF) and enrolled in the US-ALF Study Group (18), a group of 24 tertiary liver centers interested in determining etiology and outcomes of ALF. All serum samples in the US-ALF Study Group, including the one used in this study, were collected under research protocols approved by the Institutional Review Board of each of the participating centers. The presence of HBV in the sample was confirmed using primer sets Hep-1F (5´-GACTCGTGGTGGACTTCTCTCAA-3´) / Hep-2R (5´-GAAAGCCCTGCGAACCACTGAA-3´) and Hep-3F (5´-GGTGTCTTTCGGAGTGTGG-3´) / Hep-4R (5´-CGGCGATTGAGACCTTCG-3´), which were designed to amplify sequences from the genes coding for the S protein and core protein, respectively. An amplified PCR fragment was sequenced and shared >99% nucleotide identity to the genome of HBV (GenBank: NC_003977). A 100 µl frozen aliquot of the HBV sample was thawed and used to extract total nucleic acid using the QIAamp Viral Nucleic Acid Kit (Valencia, CA) according to the manufacturer’s protocol. Extracted nucleic acid was eluted in 30 µL nuclease-free water.

***Construction of initial Plasmodium falciparum cDNA library***

1.6 µg of poly-A selected RNA was fragmented in 10 mM ZnCl_2_, 10 mM TrisHCl pH 7.0 for 5 min at 70°C. The reaction was stopped with 50 mM EDTA and fragmented RNA was purified over a Zymo RNA Clean-up Kit 5 column (Zymo Research, Orange, CA), then aliquoted into a 96-well plate, each well of which contained 2.5 uM 6bp-EciI-N_9_ (all primer sequences can be found in Supplementary Table 1). After incubating the mixture at 65°C for 5 min and 25°C for 5 min, first strand cDNA synthesis mix (0.5 mM dATP, 0.5 mM dTTP, 0.125 mM dGTP, 0.125 µM dCTP, 5 mM DTT, 1x Superscript III Reverse Transcriptase First Strand buffer, 67 U Superscript III Reverse Transcriptase (Invitrogen Corp., Carlsbad, CA)) was added to each well. First strand synthesis was performed at 42°C for 1.5 hours, then stopped at 70°C for 10 min. RNA was hydrolyzed by adding 20 mM EDTA, 200 mM NaOH and incubating at 65°C for 15 min. 250 mM HEPES pH7.4 and 333 mM NaOAc pH5.2 were added to stop the reaction. First strand cDNA was purified over MinElute PCR purification columns according to the manufacture’s protocol for single stranded DNA (Qiagen Inc., Valencia, CA). Purified products were pooled, then split back out into a fresh 96-well plate for second strand cDNA synthesis.

First strand cDNA was incubated in 0.4x Sequenase buffer with 1.6 µM 13bp-ModSolS-N_9_ (Supplementary Table 1) at 80°C for 2 min, then put directly on ice. Second strand cDNA synthesis mix (0.4 mM dATP, 0.4 mM dTTP, 0.1 mM dGTP, 0.1 mM dCTP, 3.3 mM DTT, Sequenase buffer to 0.37x, and 2.86 U of Sequenase Version 2.0 DNA Polymerase (USB, Cleveland, OH)) was added to each well and synthesis was carried out by ramping from 4°C to 37°C over 8 min, then incubating at 37°C for 30 min. Second strand cDNA products were purified with AMPure PCR purification beads (Agencourt Bioscience Corp., Beverly, MA).

The pooled library was aliquoted into a 96-well plate, then PCR amplified in the following mix: 1x KlenTaq LA buffer, 0.1 mM dATP, 0.1 mM dTTP, 0.025 mM dCTP, 0.025 mM dGTP, 0.1 µM biotin-short-ModSolS, 0.1 µM 6bp-EciI, and 1 U of KlenTaq LA DNA polymerase (Sigma-Aldrich, St. Louis, MO). PCR conditions were 95°C for 2 min, then 5 cycles of 95°C for 30 sec, 52°C for 45 sec, 65°C for 3 min, and finally 65°C for 7 min. PCR products were purified with AMPure PCR purification beads, then pooled. Purified PCR products were bound to pre-washed Dynal Dynabeads M-280 (Invitrogen Corp., Carlsbad, CA) for 15 min at 25°C with gentle mixing. Bound beads were washed 3 times in 1x B&W buffer (5 mM Tris HCl pH 7.5, 0.5 mM EDTA, 1 M NaCl), then once in 0.5x NEB buffer 2, and finally were resuspended in 160 µL H_2_O and divided into 40 µL aliquots.

Bead aliquots were incubated with 2 U EciI (New England Biolabs, Ipswich, MA) in 1x NEB buffer 2 and 100 µg/mL Bovine Serum Albumin at 37°C for 1 hour, then at 65°C for 20 min to heat-inactivate the enzyme. Digestion reactions were treated with 5 U Antarctic Phosphatase (New England Biolabs, Ipswich, MA) in 1x Antarctic Phosphatase buffer at 37°C for 30 min, then at 65°C for 20 min to heat-inactivate the enzyme. Beads were washed with 1x B&W buffer, then with 0.5x T4 DNA ligase buffer and resuspended in H2O. 24bp-SolL-GsuI-NN and P-recomp24bpSolL-GsuI-6Camino were resuspended to 100 µM in 10 mM Tris pH8.0, 50 mM NaCl, 1 mM EDTA. The oligonucleotides were combined 1:1 and heated to 94°C over 5 minutes, then cooled to 4°C over 20 minutes to produce an annealed adapter (“Sol-L-NN”). Beads were incubated with 30 µM annealed adapter, 1x T4 DNA ligase buffer, and 400 cohesive end units of T4 DNA ligase (New England Biolabs, Ipswich, MA) for >12 hours at 16°C. The beads were washed with 1x B&W buffer, then with 0.5x KlenTaq LA buffer, and finally resuspended in 40 µL H_2_O. One 40 µL aliquot was removed for PCR amplification in a 96-well plate. The PCR mix and conditions were the same as above, except the fullModSolS and Sol primer 1 were used.

***Construction of initial HBV sample cDNA library***

For the HBV sample, 4 µL of extracted nucleic acid was reverse transcribed to cDNA and amplified using random primers in a modified Rd A/B protocol as previously described (Chiu, et al.*,* 2006). Briefly, after incubating the RNA at 65°C for 5 min and room temperature for 5 min, first strand cDNA synthesis mix (2.5 µM Sol-PrimerA (Supplementary Table 1), 0.125 mM of each dNTP, 5 mM DTT, 1X Superscript II Reverse Transcriptase First Strand Buffer, 67 U Superscript III Reverse Transcriptase (Invitrogen Corporation, Carlsbad, CA)) was added to the tube. First strand synthesis was performed at 42°C for 1.5 hours. The reaction products were then heated to 94°C x 2 min and cooled to 4°C. Next, second strand cDNA synthesis mix (0.125 mM of each dNTP, Sequenase buffer to 0.37x, and 2.86 U of Sequenase Version 2.0 DNA Polymerase (USB, Cleveland, OH)) was added to the tube, and second-strand synthesis carried out by ramping from 4°C to 37°C over 8 min, then incubating at 37°C for 30 min. PCR amplification was then performed in the following mix: 1X KlenTaq LA buffer, 0.125 mM of each dNTP, 1 µM Sol-PrimerB (Supplementary Table 1), and 1 U of KlenTaq LA DNA polymerase (Sigma-Aldrich, St. Louis, MO). PCR conditions were 95°C for 2 min, then 25 cycles of 95°C for 30 sec, 50°C for 1 min, 72°C for 1 min, and finally 72°C for 7 min. Afterwards, the PCR products were purified and bound to streptavidin beads as described above for *Plasmodium falciparum*, with the following modifications: (1) both ends of the PCR product were digested using GsuI instead of only one end by EciI, (2) ligation reactions were performed by adding adapters to both ends simultaneously, the first adapter (“Sol-S-RR”) constructed by annealing Sol-Adapter-S-short-phos-bio and short-SolS-RR and the second adapter (“Sol-L-AA-RR”) constructed by annealing short-SolL-GsuI-AARR and Sol-Adapter-L-short-phos-AA (Supplementary Table 1), and (3) PCR amplification of the initial aliquot was carried on for 15 additional cycles. A two-nucleotide RR overhang was used instead of NN to prevent Sol-S/Sol-L dimer formation.
