# Supplementary material for: The Long March: A Sample Preparation Technique that Enhances Contig Length and Coverage by High-Throughput Short-Read Sequencing
Source: PLoS One. 2008 Oct 22;3(10):e3495. doi: 10.1371/journal.pone.0003495 (PMC2566813; doi:10.1371/journal.pone.0003495)
Supplement: Table S1 — Primer sequences for initial library preparation and the long march. (0.02 MB DOC) [file pone.0003495.s002.doc]

Table S1. Primer sequences for initial library preparation and the long march.

| **Adapter** | **Primer** | **Primer Sequence** |
| --- | --- | --- |
|  | 6bp-EciI-N_9_ | 5´-GACGCTGGCGGANNNNNNNNN-3´ |
|  | 13bp-ModSolS-N_9_ | 5´-GCTCTGCCGCTCTNNNNNNNNN-3´ |
|  | biotin-short-ModSolS | 5´-/5Biosg/GGCATACGAGCTCTGCCGCTCT-3´ |
|  | 6bp-EciI | 5´-GACGCTGGCGGA-3´ |
|  | Sol-PrimerA | 5´-GTTTCCCACTGGAGGATANNNNNNNNN-3´ |
|  | Sol-PrimerB | 5´-GTTTCCCACTGGAGGATA-3´ |
|  | fullModSolS | 5´- CAAGCAGAAGACGGCATACGAGGCATACGAG CTCTGCCGCTCT-3´ |
|  | Sol primer 1 | 5´-AATGATACGGCGACCACCGACACTCTTTCCCTA CACGACGCTCTTCCTGGAG-3´ |
|  | Sol-SeqPrimer | 5´-CACTCTTTCCCTACACGACGCTCTTCCTGGAG-3´ |
| Sol-L-NN | 24bp-SolL-GsuI-NN | 5´-CCCTACACGACGCTCTTCCTGGAGNN-3´ |
|  | P-recomp24bpSolL-GsuI-6Camino | 5´-/5Phos/CTCCAGGAAGAGCGTCGTGTAGGG /3AmM /-3´ |
| Sol-L-AA-NN | short-SolL-GsuI-AANN | 5´-CACGACGCTCTTCCTGGAGAANN-3´ |
|  | Sol-Adapter-L-short-phos-AA | 5´-/5Phos/TTCTCCAGGAAGAGCGTCGTG/3AmM/-3´ |
| Sol-L-CC-NN | short-SolL-GsuI-CCNN | 5´-CACGACGCTCTTCCTGGAGCCNN-3´ |
|  | Sol-Adapter-L-short-phos-CC | 5´-/5Phos/GGCTCCAGGAAGAGCGTCGTG/3AmM/-3´ |
| Sol-L-TT-NN | short-SolL-GsuI-TTNN | 5´-CACGACGCTCTTCCTGGAGTTNN-3´ |
|  | Sol-Adapter-L-short-phos-TT | 5´-/5Phos/AACTCCAGGAAGAGCGTCGTG/3AmM/-3´ |
| Sol-S-RR | short-SolS-RR | 5´-GCATACGAGCTTTCCGATCTRR-3´ |
|  | Sol-Adapter-S-short-phos-bio | 5´-/5Phos/5Biosg/AGATCGGAAGAGCTCGTATGC /3AmM/-3´ |
| Sol-L-AA-RR | short-SolL-GsuI-AARR | 5´-CACGACGCTCTTCCTGGAGAARR-3´ |
|  | Sol-Adapter-L-short-phos-AA | 5´-/5Phos/TTCTCCAGGAAGAGCGTCGTG/3AmM/-3´ |
| Sol-L-CC-RR | short-SolL-GsuI-CCRR | 5´-CACGACGCTCTTCCTGGAGCCRR-3´ |
|  | Sol-Adapter-L-short-phos-CC | 5´-/5Phos/GGCTCCAGGAAGAGCGTCGTG/3AmM/-3´ |
| Sol-L-GG-RR | short-SolL-GsuI-GGRR | 5´-CACGACGCTCTTCCTGGAGGGRR-3´ |
|  | Sol-Adapter-L-short-phos-GG | 5´-/5Phos/CCCTCCAGGAAGAGCGTCGTG/3AmM/-3´ |
| Sol-L-TT-RR | short-SolL-GsuI-TTRR | 5´-CACGACGCTCTTCCTGGAGTTRR-3´ |
|  | Sol-Adapter-L-short-phos-TT | 5´-/5Phos/AACTCCAGGAAGAGCGTCGTG/3AmM/-3´ |
